# Screening the Potential Biomarkers of COVID-19-Related Thrombosis Through Bioinformatics Analysis

**DOI:** 10.3389/fgene.2022.889348

**Published:** 2022-05-25

**Authors:** Peng Qi, Mengjie Huang, Tanshi Li

**Affiliations:** ^1^ Department of Emergency, First Medical Center of Chinese PLA General Hospital, Beijing, China; ^2^ Department of Nephrology, First Medical Center of Chinese PLA General Hospital, Beijing, China

**Keywords:** COVID-19, thrombosis, therapy, bioinformatic analysis, differentially expressed genes

## Abstract

A high proportion of critically ill patients with coronavirus disease 2019 (COVID-19) experience thrombosis, and there is a strong correlation between anticoagulant therapy and the COVID-19 survival rate, indicating that common COVID-19 and thrombosis targets have potential therapeutic value for severe COVID-19.Gene expression profiling data were downloaded from Gene Expression Omnibus (GEO), and common differentially expressed genes (co-DEGs) were identified. The potential biological functions of these co-DEGs were explored by functional enrichment analysis, and protein–protein interaction (PPI) networks were constructed to elucidate the molecular mechanisms of the co-DEGs. Finally, hub genes in the co-DEG network were identified, and correlation analysis was performed.We identified 8320 upregulated genes and 7651 downregulated genes from blood samples of COVID-19 patients and 368 upregulated genes and 240 downregulated genes from blood samples of thrombosis patients. The enriched cellular component terms were mainly related to cytosolic ribosomes and ribosomal subunits. The enriched molecular function terms were mainly related to structural constituents of ribosomes and electron transfer activity. Construction of the PPI network and identification of hub genes ultimately confirmed that RPS7, IGF1R, DICER1, ERH, MCTS1, and TNPO1 were jointly upregulated hub genes, and FLNA and PXN were jointly downregulated hub genes.The identification of novel potential biomarkers provides new options for treating COVID-19-related thrombosis and reducing the rate of severe COVID-19.

## Introduction

The coronavirus disease 2019 (COVID-19) pandemic has had a significant impact on global public health, social interactions and the economy (Han et al., 2020; Global Burden of Disease 2020 Health Financing Collaborator Network, 2021; Lal et al., 2021; Mcintyre et al., 2022). As of February 2022, nearly 400 million people have been diagnosed with COVID-19 worldwide, and more than 5.7 million people have died. COVID-19 can induce different complications and affect different organs (Drake et al., 2021; Wang et al., 2021), mainly the respiratory tract, heart, gastrointestinal tract (Serban et al., 2021; Bulte et al., 2022), central nervous system (Sriwastava et al., 2021), liver, and kidney (Gross et al., 2020). Elderly individuals and patients with underlying diseases are prone to severe cases, and their mortality rates are significantly higher than those of other populations (Ghasemiyeh et al., 2021). Patients who die from COVID-19 infection in the late stage of disease progression can develop acute respiratory distress syndrome (ARDS) (Zangrillo et al., 2022), cytokine storm syndrome (CSS) (Yongzhi, 2021), acute kidney injury (AKI) (Ronco et al., 2020), septic shock (Wei et al., 2021), multiple organ failure (MOF) (Devaux et al., 2020) and cardiac arrest (Shao et al., 2020), resulting in death. Especially in patients with severe COVID-19, cardiac manifestations such as arrhythmia, myocardial infarction, acute heart failure and cardiogenic shock are common and are associated with increased mortality (Bangalore et al., 2020; Lala et al., 2020). Researchers have proposed various hypotheses about the direct and indirect mechanisms of COVID-19-related heart damage, but the exact mechanism is still unclear (Chung et al., 2021). The earlier theory that COVID-19 heart damage is due to acute viral myocarditis has been disproved by the lack of cases meeting the histological criteria for myocarditis (Halushka and Vander Heide, 2021). Subsequently, further studies have suggested that coagulation abnormalities, including thrombotic microangiopathy and complications of venous and arterial thromboembolism, are common in critically ill patients with COVID-19 (Levi et al., 2020). The pathological results suggest that the most common pathological cause of myocardial cell necrosis is the occurrence of microthrombi (Pellegrini et al., 2021). Moreover, a series of studies have also found that severe acute respiratory syndrome coronavirus 2 (SARS-CoV-2) infection promotes hypercoagulability, and there is a strong correlation between the use of anticoagulants and COVID-19 survival (Nadkarni et al., 2020; Fröhlich et al., 2021; Lawler et al., 2021). A series of studies have confirmed that COVID-19 is associated with venous thromboembolic events, including pulmonary embolism, lower extremity deep venous thrombosis and cerebral venous sinus thrombosis, and there is a correlation between thrombosis and mortality in patients with COVID-19 (Rosovsky et al., 2020). Some studies have even found a 51.5-fold increased risk of in-hospital mortality in patients with D-2mer ≥2.0 μg/ml (Zhang L. et al., 2020), but the actual prevalence and characteristics of venous thromboembolism (VTE) remain unclear (Sastry et al., 2022). The incidence of VTE reported by different studies varies significantly (ranging from 0.7% to 57%) due to differences in disease severity and diagnostic imaging techniques used (Cui et al., 2020; Klok et al., 2020; Lodigiani et al., 2020; Suh et al., 2021). Even with anticoagulant prophylaxis, the incidence of VTE in COVID-19 patients remains high and is much higher than that in non-COVID patients receiving thromboprophylaxis (Lodigiani et al., 2020). Cytokine storms were initially believed to be the main factor for COVID-19 thrombosis (Lowenstein and Solomon, 2020), antiphospholipid antibodies were later detected in COVID-19 (Zhang Y. et al., 2020; Zuo et al., 2020), and the presence of prothrombotic antibodies also constituted one of the risk factors. Studies have also suggested that elevated levels of neutrophil extracellular traps are closely related to thrombotic events (Zuo et al., 2021), but their overall role in COVID-19-related thrombosis remains unclear (Bonaventura et al., 2021). At present, most reports on thrombosis in patients with COVID-19 are case reports (Achim et al., 2022; Bleiziffer et al., 2022; Jeckelmann et al., 2022; Russell et al., 2022; Takasu et al., 2022) and some observational studies (Maier et al., 2020). Even after strict ethical review, only a small number of large studies have confirmed the relationship between the use of anticoagulants and the survival rate of COVID-19 (Lawler et al., 2021). There is a good correlation. Therefore, it is still necessary to obtain a theoretical basis for the mechanism of COVID-19 thrombosis. In summary, exploring targets that can be used to treat both COVID-19 and thrombosis has theoretical guiding significance for the assessment and treatment of COVID-19 patients. However, there are various difficulties in clinical research on COVID-19, including ethical requirements and limited patient contact. The use of public databases solves the above problems. Further analysis of relevant datasets from public databases we can be used to identify and develop reliable biomarkers and treatment strategies for thrombosis in patients with COVID-19 and thus to better understand the mechanism of thrombosis in patients with COVID-19 and identify the corresponding targets. Such studies will promote follow-up drug research and development. We identified 8320 upregulated genes and 7651 downregulated genes from blood samples of COVID-19 patients and 368 upregulated genes and 240 downregulated genes from blood samples of thrombosis patients. We identified hub genes that may contribute to the evaluation and treatment of COVID-19-related thrombosis.

## Materials and Methods

### Data Collection and Processing

The GEO database was searched with “COVID-19” and “thrombosis” as keywords, and we searched using keywords and restricted the screening conditions to “*Homo sapiens*” and “Expression profiling by array” and limited the sample to peripheral blood. After reading the original studies on the data sets that met the screening conditions individually, we focused on the experimental design and methodology. The data set that met the principle of randomized control and did not violate ethical guidelines was taken as the research object. After screening each dataset, GSE19151 and GSE164805 were ultimately selected for further research. The GSE19151 dataset included 70 adults who had used warfarin one or more times for venous thromboembolism and 63 healthy controls. This dataset excluded patients with antiphospholipid syndrome and cancer and was generated using the GPL571 [HG-U133A_2] Affymetrix Human Genome U133A 2.0 Array. The GSE164805 dataset included 10 patients diagnosed with COVID-19 and 5 healthy controls and was generated using the GPL26963 Agilent-085982 Arraystar Human LncRNA V5 microarray. R software (version 4.0.2, http://r-project.org/) was used to download the two datasets and relevant information. The raw data were processed through the “affy” (Gautier et al., 2004) package, and the RMA algorithm was used to perform background correction and data normalization. The normalized results were visualized using boxplots (Supplementary Figures S1, S2).

### Identification of DEGs

The “limma” package of R software was used to analyze the disease group and healthy control group of the two datasets, and the differentially expressed genes (DEGs) between the two groups were obtained. To identify the most meaningful DEGs, the following limits were used to determine which DEGs would be retained: (1) | log2 (fold-change) | > 0.5 and (2) adjusted *p* < 0.05. In addition, adjusted *p* < 0.05 was considered to indicate a significant difference (Ritchie et al., 2015; Shankar et al., 2020).

### GO and KEGG Pathway Enrichment Analysis of the DEGs

Gene Ontology (GO) functional analysis (including cellular component [CC], biological process [BP], and molecular function [MF] analysis) is a powerful bioinformatics tool for classifying gene expression and associated properties (Ashburner et al., 2000). Kyoto Encylopedia of Genes and Genomes (KEGG) pathway analysis was used to determine which cellular pathways may be related to the DEGs (Altermann and Klaenhammer, 2005). The “clusterProfiler" (Yu et al., 2012) package of R software was used for enrichment analysis of the DEGs, the “org.Hs.eg.db” package was used for ID conversion, and the “Goplot” package was used to calculate Z scores (Walter et al., 2015). The results are visually presented as chord graphs and tables.

### Identification of Co-DEGs and Construction of the PPI Network

The co-DEGs of the two datasets were analyzed using a Venn diagram, and the results were visualized using the “ggplot2” (Rosenthal et al., 2019) package of R software. The co-upregulated genes and co-downregulated genes were subsequently uploaded to the Search Tool for the Retrieval of Interacting Genes/Proteins (STRING, http://string-db.org/) database for protein–protein interaction (PPI) network construction, and 0.4 was used as the minimum required interaction score for analysis. The results were input into Cytoscape (Shannon et al., 2003) to construct and visualize the molecular interaction network.

### Identification of Hub Genes

Hub genes are the DEGs that show the highest association with other network components, and they are important nodes in the PPI network. The Cyto-Hubba (Chin et al., 2014) plug-in in Cytoscape software was used to determine the hub genes in the PPI network. To reduce the selection bias caused by different algorithms, the MCC, DMNC, MNC, and EPC algorithms were used to determine the hub genes, and the final results were confirmed and visualized with a Venn diagram.

### Functional Enrichment Analysis of Hub Genes

The selected hub genes were analyzed by the PANTHER(Mi et al., 2019) classification system (http://pantherdb.org/), and the basic classification of each gene was obtained. Then, the Human Protein Atlas (Uhlen et al., 2019) (HPA) (https://www.proteinatlas.org/) was used to explore the expression profile of each hub gene in human immune cells. Subsequently, the Deeply Integrated human Single-Cell Omics (DISCO) database (Li et al., 2022) (https://www.immunesinglecell.org/) was used to clarify the expression level of the hub genes in all blood cells of COVID-19 patients. Finally, the “clusterProfiler” (Yu et al., 2012) package of R software was used for the enrichment analysis of the hub genes, and the “ggplot2” package of R software was used to visually present the results of the enrichment analysis. The results are displayed in a histogram and table.

## Results

### Identification of DEGs

**FIGURE 1:**
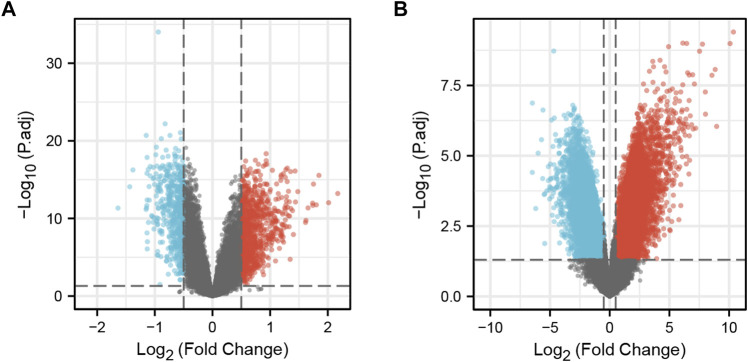
DEGs in the two datasets. **(A)** Volcano plot of GSE19151; Red represents upregulated genes, blue represents downregulated genes, and gray represents no significantly expressed genes. **(B)** Volcano plot of GSE164805. The criteria for statistically significant difference of DEGs was adjusted |log2(FC)|>0.5 and p.adj<0.05 in expression.

The “limma” package of R software was used to identify the DEGs of the two datasets. In the GSE19151 dataset, included 70 adults who had used warfarin one or more times for venous thromboembolism and 63 healthy controls, the number of genes after data processing was 21144, including 368 upregulated genes that met the log2 (FC) > 0.5 and adjusted *p* < 0.05 criteria and 240 downregulated genes that met the log2 (FC) < −0.5 and adjusted *p* < 0.05 criteria. In the GSE164805 dataset, included 10 patients diagnosed with COVID-19 and 5 healthy controls, the number of genes after data processing was 60494, including 8320 upregulated genes meeting the log2 (FC) > 0.5 and adjusted *p* < 0.05 criteria and 7651 downregulate genes meeting the log2 (FC) < −0.5 and adjusted *p* < 0.05 criteria. A volcano plot was generated using the “ggplot2” package to visualize the DEGs (Figure 1).

### GO and KEGG Pathway Enrichment Analyses of DEGs

**FIGURE 2:**
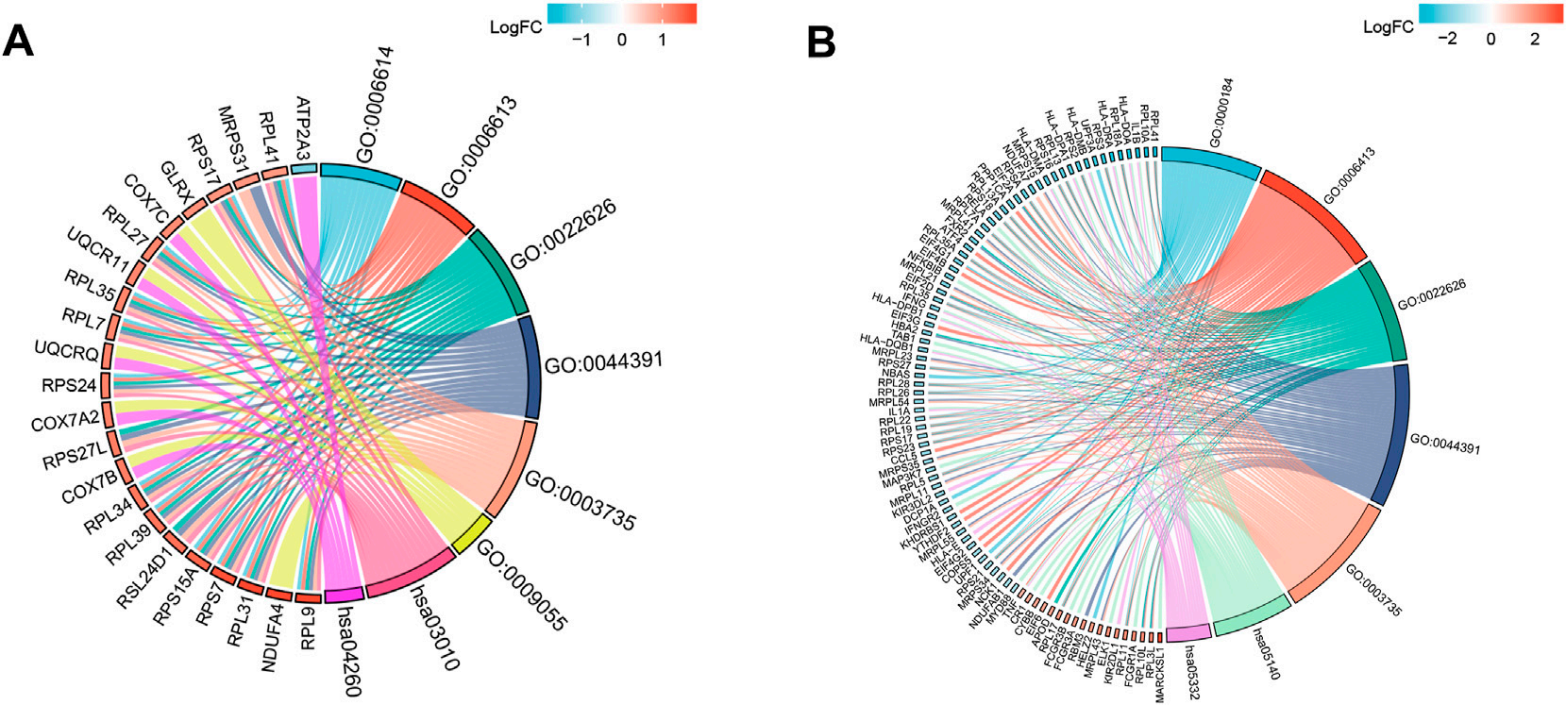
GO terms and KEGG pathway enrichment. **(A)** Details of GO terms and KEGG pathway enrichment in GSE19151; The left side of the figure indicated that the genes were sorted according to logFC. Red represented up-regulation, blue represented down-regulation, and color depth represented change multiples. The right side of the figure indicates the order of enrichment analysis term according to the change of strength. There is a link between genes and term, indicating that they have a subordinate relationship. **(B)** Details of GO terms and KEGG pathway enrichment in GSE164805.

The main enriched BP terms of the DEGs in the venous thrombosis dataset were SRP-dependent cotranslational protein targeting to membrane (GO:0006614) and cotranslational protein targeting to membrane (GO:0006613). The main enriched CC terms were cytosolic ribosome (GO:0022626) and ribosomal subunit (GO:0044391), and the main enriched MF terms were structural constituent of ribosome (GO:0003735) and electron transfer activity (GO:0009055). The KEGG pathway analysis mainly showed enrichment of the terms ribosome (hsa03010) and cardiac muscle contraction (hsa04260). For the DEGs identified from the COVID-19 dataset, the enriched BP terms mainly included nuclear-transcribed mRNA catabolic process, nonsense-mediated decay (GO:0000184) and translational initiation (GO:0006413), the enriched CC terms mainly included cytosolic ribosome (GO:0022626) and ribosomal subunit (GO:0044391), and the enriched MF term was structural constituent of ribosome (GO:0003735). The KEGG pathway enrichment analysis mainly identified the terms leishmaniasis (hsa05140) and graft-versus-host disease as enriched (hsa05332). For both datasets, the CC and MF terms were similar. All the results are presented in chord diagrams (Figure 2) and tables (Tables 1, 2).

**TABLE 1:** Details of GO terms and KEGG pathway enrichment in GSE19151.

Ontology	ID	Description	GeneRatio	BgRatio	*p* value	p.adjust	q value
BP	GO:0006614	SRP-dependent cotranslational protein targeting to membrane	12/99	105/18670	3.24e-13	5.61e-10	4.88e-10
BP	GO:0006613	cotranslational protein targeting to membrane	12/99	109/18670	5.11e-13	5.61e-10	4.88e-10
CC	GO:0022626	cytosolic ribosome	14/102	112/19717	6.19e-16	1.70e-13	1.52e-13
CC	GO:0044391	ribosomal subunit	15/102	190/19717	5.51e-14	7.55e-12	6.76e-12
MF	GO:0003735	structural constituent of ribosome	15/98	202/17697	3.43e-13	1.02e-10	9.46e-11
MF	GO:0009055	electron transfer activity	7/98	114/17697	3.26e-06	4.83e-04	4.50e-04
KEGG	hsa03010	Ribosome	14/56	158/8076	1.93e-12	3.16e-10	2.88e-10
KEGG	hsa04260	Cardiac muscle contraction	6/56	87/8076	2.77e-05	0.002	0.002

**TABLE 2:** Details of GO terms and KEGG pathway enrichment in GSE164805.

Ontology	ID	Description	GeneRatio	BgRatio	pvalue	p.adjust	q value
BP	GO:0000184	nuclear-transcribed mRNA catabolic process, nonsense-mediated decay	30/1653	120/18670	1.28e-07	7.54e-04	7.17e-04
BP	GO:0006413	translational initiation	39/1653	193/18670	8.02e-07	0.002	0.002
CC	GO:0022626	cytosolic ribosome	31/1734	112/19717	5.12e-09	3.58e-06	3.31e-06
CC	GO:0044391	ribosomal subunit	42/1734	190/19717	1.80e-08	6.29e-06	5.83e-06
MF	GO:0003735	structural constituent of ribosome	37/1638	202/17697	4.24e-05	0.045	0.044
KEGG	hsa05140	Leishmaniasis	24/774	77/8076	1.00e-07	3.15e-05	2.69e-05
KEGG	hsa05332	Graft-versus-host disease	14/774	42/8076	2.01e-05	0.003	0.003

### PPI Network Construction and Hub Gene Identification

**FIGURE 3:**
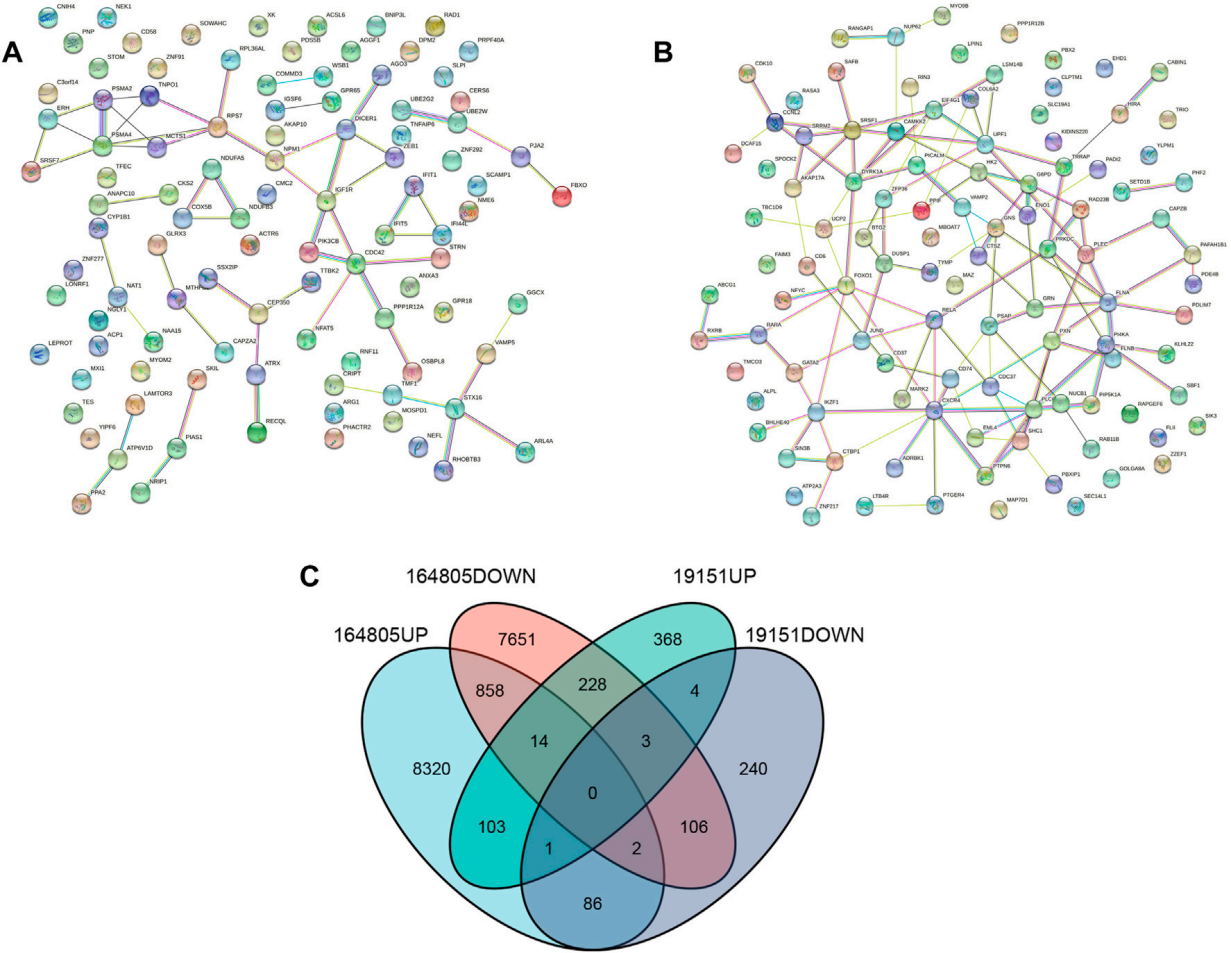
PPI network and Venn diagram. **(A)** A PPI network were constructed by co-upregulated genes. **(B)** A PPI network were constructed by co-downregulated genes. **(C)** The Venn diagram showed the co-DEGs of the two datasets.

**FIGURE 4:**
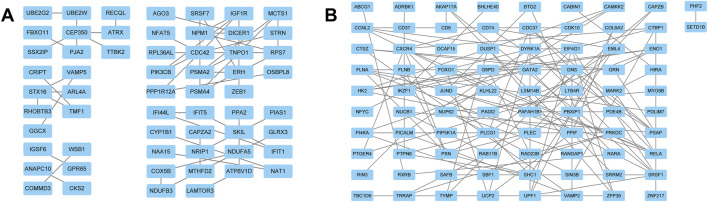
PPI network of DEGs. **(A)** The PPI network of the co-upregulated genes included 59 nodes and 57 edges. **(B)** The PPI network of the co-downregulated genes included 82 nodes and 126 edges.

**FIGURE 5:**
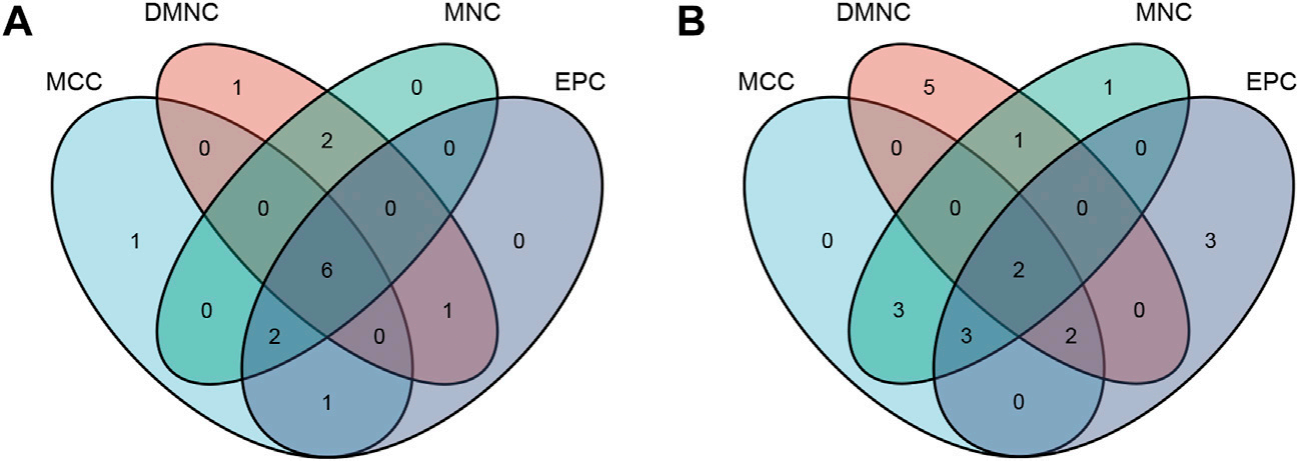
The hub genes were merged by the MCC, DMNC, MNC, and EPC algorithms. **(A)** RPS7, IGF1R, DICER1, ERH, MCTS1, and TNPO1 were jointly upregulated hub genes. **(B)** FLNA and PXN were identified as jointly downregulated hub genes.

The “VennDiagram” package of R software was used to extract the co-DEGs of the two datasets (Figure 3C). A total of 103 co-upregulated genes and 106 co-downregulated genes were identified. The co-upregulated genes and co-downregulated genes were uploaded to the STRING database to construct a PPI network (Figures 3A,B), and the results were imported into Cytoscape software for further analysis. The PPI network of the co-upregulated genes included 59 nodes and 57 edges, and the PPI network of the co-downregulated genes included 82 nodes and 126 edges (Figure 4). Next, the Cyto-Hubba plugin was used to identify the hub genes. To reduce the selection bias caused by different algorithms, the MCC, DMNC, MNC, and EPC algorithms were used to extract the hub genes, and the “VennDiagram” package was used to merge the hub genes extracted by the four algorithms (Figure 5). Ultimately, RPS7, IGF1R, DICER1, ERH, MCTS1, and TNPO1 were identified as jointly upregulated hub genes, and FLNA and PXN were identified as jointly downregulated hub genes.

### Analysis of Hub Genes

**FIGURE 6:**
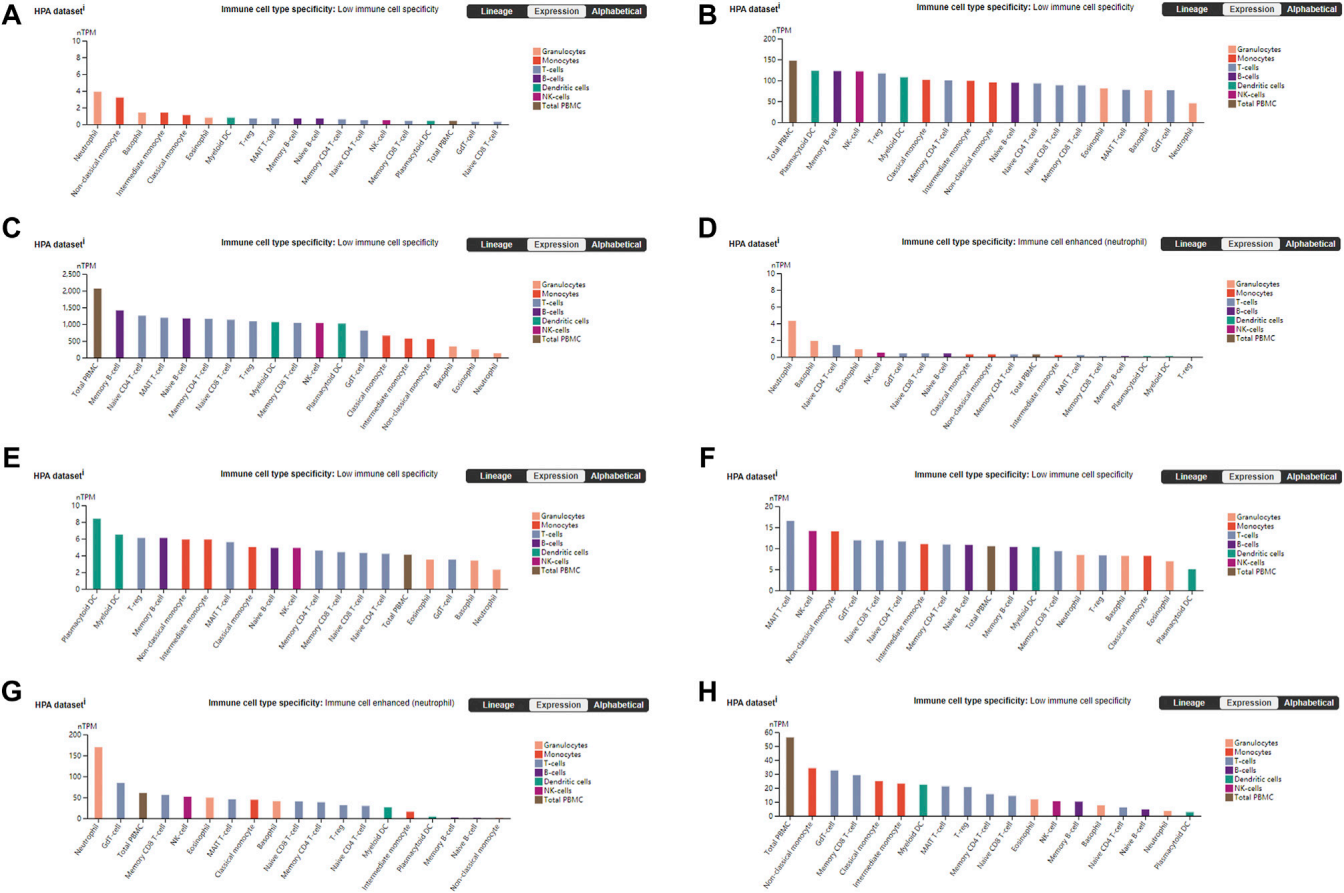
Blood cell type expression overview shows RNA-seq data from internally generated Human Protein Atlas (HPA) data. Color-coding is based on blood cell type lineages. The resulting transcript expression values calculated as nTPM, resulting from the internal normalization pipeline for 18 immune cell types and total PBMC. **(A)** DICER1 **(B)** ERH **(C)** RPS7 **(D)** IGF1R **(E)** MCTS1 **(F)** TNP01 **(G)** PXN **(H)** FLNA.

**FIGURE 7:**
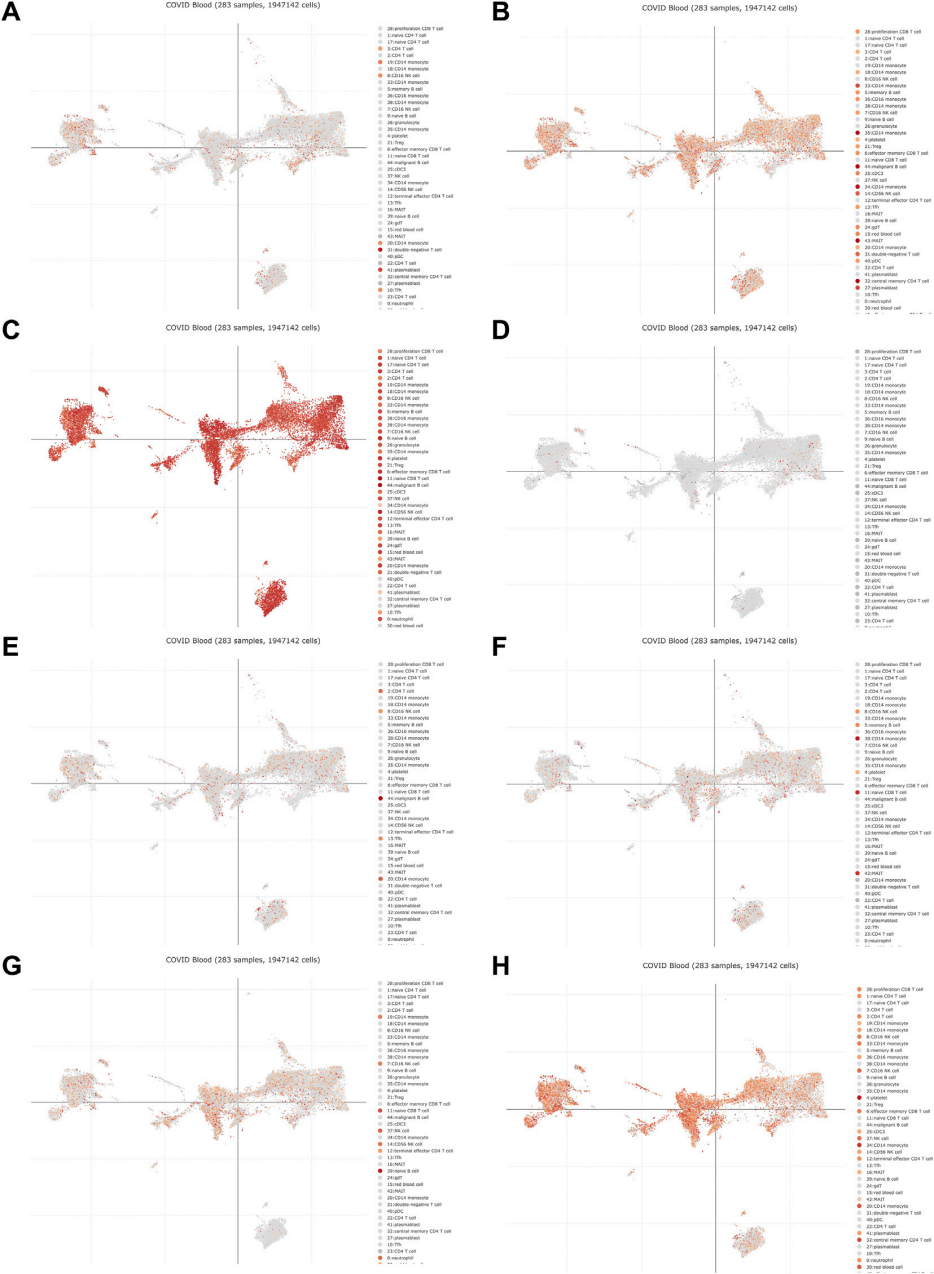
The DISCO database was used annotate the single-cell sequencing data of blood cells from patients with COVID-19. The deeper the color is, the higher the expression of the gene in the cell. **(A)** DICER1 **(B)** ERH **(C)** RPS7 **(D)** IGF1R **(E)** MCTS1 **(F)** TNP01 **(G)** PXN **(H)** FLNA.

The PANTHER (Mi et al., 2019) classification system was used to analyze the selected hub genes, and the basic classification of each gene was obtained. The results are listed in a table (Table 3). Complex interactions between innate immunity, platelet activation and coagulation lead to immune thrombosis. Immune thrombosis is an important host defense mechanism that limits the systematic transmission of pathogens through blood flow. However, abnormal activation of immune thrombosis can also lead to myocardial infarction, stroke and venous thromboembolism (Stark and Massberg, 2021). To explore the expression of the hub genes in human immune cells, the HPA database was used to analyze each hub gene (Figure 6). HPA RNA-seq data are displayed in different colors based on cell type, including B cells, T cells, natural killer (NK) cells, monocytes, granulocytes, dendritic cells and total peripheral blood mononuclear cells (PBMCs). The DISCO (Li et al., 2022) database was used annotate the single-cell sequencing data of blood cells from patients with COVID-19. The expression level of each hub gene in each cell is shown. The deeper the color is, the higher the expression of the gene in the cell (Figure 7).

**TABLE 3:** The PANTHER classification system of Hub genes.

ID	Gene name	Gene ID	PANTHER Family/Subfamily	PANTHER Protein Class
MCTS1	Malignant T-cell-amplified sequence 1	HUMAN|HGNC = 23357|UniProtKB = Q9ULC4	MALIGNANT T-CELL-AMPLIFIED SEQUENCE 1 (PTHR22798:SF12)	translation initiation factor
DICER1	Endoribonuclease Dicer	HUMAN|HGNC = 17098|UniProtKB = Q9UPY3	ENDORIBONUCLEASE DICER (PTHR14950:SF37)	—
ERH	Enhancer of rudimentary homolog	HUMAN|HGNC = 3447|UniProtKB = P84090	ENHANCER OF RUDIMENTARY HOMOLOG (PTHR12373:SF9)	DNA-binding transcription factor
TNPO1	Transportin-1	HUMAN|HGNC = 6401|UniProtKB = Q92973	TRANSPORTIN-1 (PTHR10527:SF21)	transporter
RPS7	40S ribosomal protein S7	HUMAN|HGNC = 10440|UniProtKB = P62081	40S RIBOSOMAL PROTEIN S7 (PTHR11278:SF5)	ribosomal protein
IGF1R	Insulin-like growth factor 1 receptor	HUMAN|HGNC = 5465|UniProtKB = P08069	INSULIN-LIKE GROWTH FACTOR 1 RECEPTOR (PTHR24416:SF106)	transmembrane signal receptor
PXN	Paxillin	HUMAN|HGNC = 9718|UniProtKB = P49023	PAXILLIN (PTHR24216:SF11)	actin or actin-binding cytoskeletal protein
FLNA	Filamin-A	HUMAN|HGNC = 3754|UniProtKB = P21333	FILAMIN-A (PTHR38537:SF5)	—

### Functional Enrichment Analysis of Hub Genes

**FIGURE 8:**
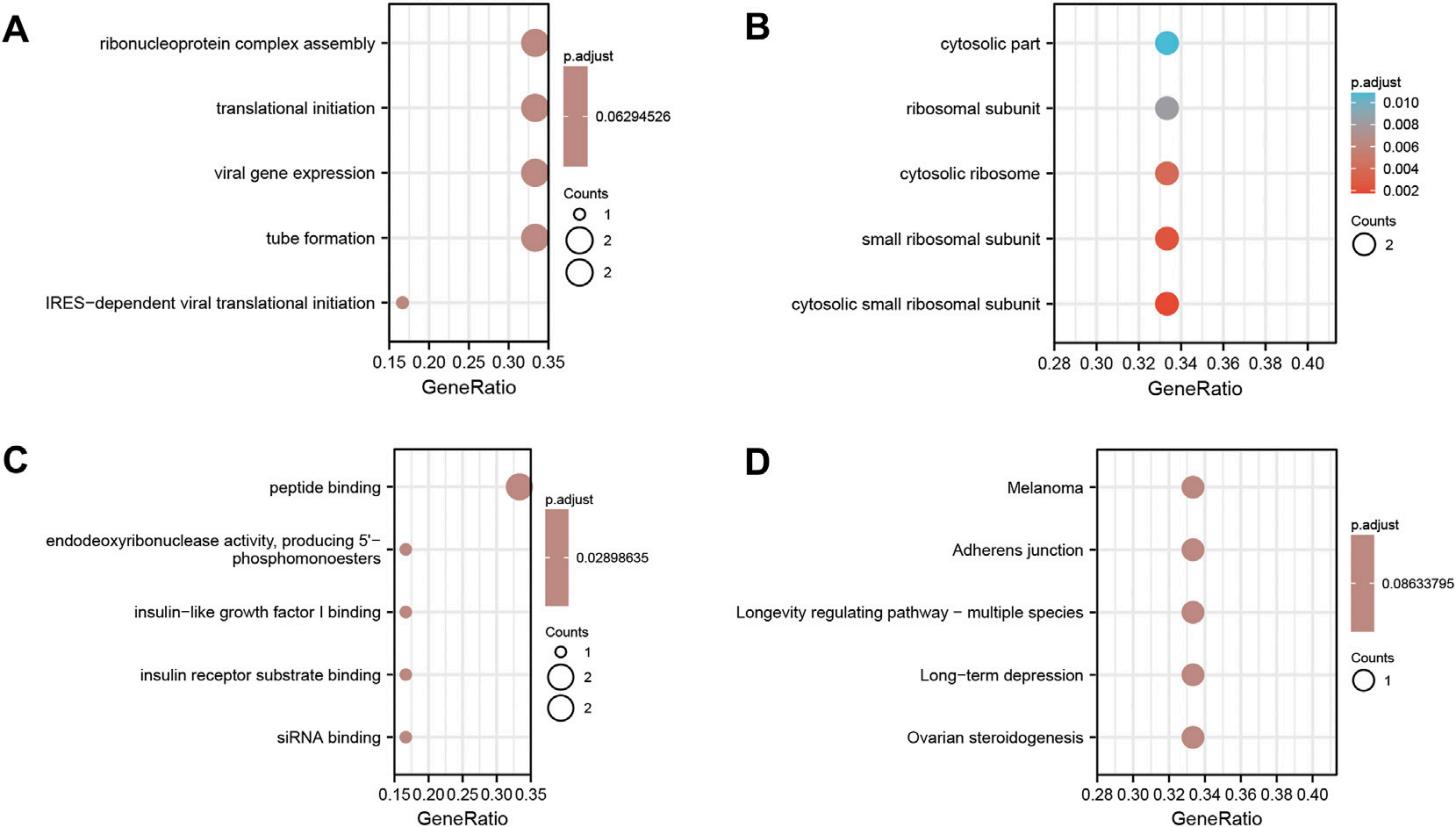
Functional enrichment analysis of the six co-upregulated hub genes. **(A)** Shows the results of biological process terms enriched by BP analysis; **(B)** Shows the results of biological process terms enriched by CC analysis; **(C)** Shows the results of biological process terms enriched by MF analysis; **(D)** Shows the enriched pathway by KEGG analysis. The coloured dots represent the *p*-value for that term, with red representing greater significance. The size of the dots represents the number of involved genes.

Functional enrichment analysis of the six coupregulated hub genes (RPS7, IGF1R, DICER1, ERH, MCTS1, and TNPO1) was performed. The results showed that the main enriched BP terms were the ribonucleoprotein complex assembly (GO: 0022618), viral gene expression (GO: 0019080) and translational initiation (GO: 0006413). The main enriched CC terms were the cytosolic part (GO: 0044445) and ribosomal subunit (GO: 0044391), and the main enriched MF term was peptide binding (GO: 0042277). The KEGG pathway analysis mainly showed enrichment of the terms adherens junction (hsa04520), melanoma (hsa05218) and longevity regulating pathway - multiple species (hsa04213). All results are displayed in a bubble diagram (Figure 8) and table (Table 4).

**TABLE 4:** Details of GO terms and KEGG pathway enrichment in co-upregulated Hub genes.

Ontology	ID	Description	GeneRatio	BgRatio	p value	p.adjust	q value
BP	GO:0035148	tube formation	2/6	148/18670	9.17e-04	0.063	0.029
BP	GO:0019080	viral gene expression	2/6	191/18670	0.002	0.063	0.029
BP	GO:0006413	translational initiation	2/6	193/18670	0.002	0.063	0.029
BP	GO:0022618	ribonucleoprotein complex assembly	2/6	277/18670	0.003	0.063	0.029
BP	GO:0075522	IRES-dependent viral translational initiation	1/6	10/18670	0.003	0.063	0.029
CC	GO:0022627	cytosolic small ribosomal subunit	2/6	44/19717	7.26e-05	0.002	7.64e-04
CC	GO:0015935	small ribosomal subunit	2/6	72/19717	1.95e-04	0.002	0.001
CC	GO:0022626	cytosolic ribosome	2/6	112/19717	4.73e-04	0.004	0.002
CC	GO:0044391	ribosomal subunit	2/6	190/19717	0.001	0.008	0.004
CC	GO:0044445	cytosolic part	2/6	247/19717	0.002	0.011	0.005
MF	GO:0035197	siRNA binding	1/6	11/17697	0.004	0.029	0.005
MF	GO:0043560	insulin receptor substrate binding	1/6	11/17697	0.004	0.029	0.005
MF	GO:0042277	peptide binding	2/6	295/17697	0.004	0.029	0.005
MF	GO:0031994	insulin-like growth factor I binding	1/6	12/17697	0.004	0.029	0.005
MF	GO:0016888	endodeoxyribonuclease activity, producing 5′-phosphomonoesters	1/6	16/17697	0.005	0.029	0.005
KEGG	hsa04913	Ovarian steroidogenesis	1/3	51/8076	0.019	0.086	0.045
KEGG	hsa04730	Long-term depression	1/3	60/8076	0.022	0.086	0.045
KEGG	hsa04213	Longevity regulating pathway - multiple species	1/3	62/8076	0.023	0.086	0.045
KEGG	hsa04520	Adherens junction	1/3	71/8076	0.026	0.086	0.045
KEGG	hsa05218	Melanoma	1/3	72/8076	0.027	0.086	0.045

### Verification of Hub Genes

To explore whether the hub genes of COVID-19 and thrombosis are common targets caused by viral induction, we included a third-party data set for corresponding verification. Previous studies have confirmed that viral diseases induce platelet and neutrophil recruitment to the microvascular system to protect host cells from viral infection (Jenne et al., 2013). However, recent research has also shown that compared with that in patients with COVID-19 pneumonia, immunothrombotic vessel occlusion in the lungs is less prominent in patients with influenza pneumonia (Ackermann et al., 2020; Nicolai et al., 2021). We searched the GEO database with “SARS” as the key word, searched using keywords, restricted the screening conditions to “*Homo sapiens*” and “Expression profiling by array” and limited the sample to peripheral blood. After reading the original studies on the data sets that met the screening conditions individually, we focused on the experimental design and methodology. The data set that met the principle of randomized control and did not violate ethical guidelines was taken as the research object. Finally, GSE5972 was selected for further research. The GSE5972 data set included 50 SARS patients, regardless of age, sex or past medical history. Positive PCR or serum results confirmed that each patient was infected with SARS-CoV. Ten healthy volunteers, five men and five women, were recruited. The chip platform was GPL4387 UHNMAC *Homo sapiens* 19K Hu19Kv8. Using the aforementioned methodology, we performed background correction and data normalization on the GSE5972 data set and then used the “limma” package of R software to analyze the disease group and the healthy control group to identify DEGs. The inclusion criteria of DEGs were the same as those for the above two data sets. The DEGs of the GSE5972 and GSE19151 data sets were combined and analyzed, the co-DEGs were screened out, and a PPI network was constructed. Then, the hub genes were extracted by Cytoscape software. The final results showed that the upregulated hub genes were IFI44L, TNFSF10, RSAD2, ETFA and MDH1 and that the downregulated hub genes were PXN, MYH9, SPTAN1, TLN1, CXCR4, SUPT6H and ENO1. It can be seen from the results that the hub genes of COVID-19 and thrombosis are not the same as the hub genes of SARS-CoV infection and thrombosis, which also explains why there are many studies on the mechanism of virus-induced thrombosis (Boilard et al., 2014; Tripathi et al., 2014; Koupenova et al., 2019) but the specific mechanism of COVID-19 thrombosis is still unclear (Farkouh et al., 2022; Thomas and Scully, 2022; Vincent et al., 2022). In addition, there have been an increasing number of recent reports of thrombosis after COVID-19 vaccination (De Michele et al., 2022), all of which indicate the existence of characteristic factors of thrombosis in COVID-19, which deserves further exploration.

## Discussion

Thrombosis and the inflammatory response were once thought to be two independent physiological processes, but in recent years, studies have gradually found that there is a clear correlation between them. Activation of the coagulation system is part of the host response to pathogens and primarily serves to limit their spread in the bloodstream, which is achieved through interactions between innate immune cells and platelets, termed immunothrombosis (Engelmann and Massberg, 2013). Due to the COVID-19 pandemic, this normal physiological response has received more attention (Stark and Massberg, 2021). Histological analysis of pulmonary vessels in COVID-19 patients showed extensive thrombosis with microangiopathy, and the incidence of pulmonary capillary microthrombosis in COVID-19 patients was 9 times higher than that in influenza patients; the difference was statistically significant (Ackermann et al., 2020). Furthermore, data show that immune thrombosis is also a key indicator of the severity of COVID-19 (Nicolai et al., 2020). Preliminary studies in Wuhan, China, showed that most patients with COVID-19 who died of disease had coagulation disorders, characterized by prolonged prothrombin time and partial prothrombin kinase time, similar to the characteristics observed in patients with disseminated intravascular coagulation (Zhou et al., 2020). When pathogens invade the bloodstream and pose a lethal threat to the host, the body tries to control the infection by all available means, and viral diseases induce the recruitment of platelets and neutrophils to the microvasculature to protect host cells from viral infection (Jenne et al., 2013). The main cellular drivers of this process are platelets and innate immune cells, including neutrophils, monocytes and macrophages. In general, the response to viral disease should be similar; however, immune thrombotic vaso-occlusion was less prominent in patients with influenza-related pneumonia than in patients with COVID-19 (Nicolai et al., 2021). This finding illustrates that the immunothrombosis in COVID-19 is not just a general feature of viral infections but may also be a characteristic factor, suggesting that it is worthy of further exploration to reduce the disease severity and mortality of patients with COVID-19. Bioinformatics analysis enables us to understand the molecular mechanisms of disease initiation and progression, providing a new and effective method for identifying potential biomarkers and therapeutic targets for assessing and treating COVID-19 thrombosis. In this study, six co-upregulated genes, RPS7, IGF1R, DICER1, ERH, MCTS1, and TNPO1, and two co-downregulated genes, FLNA and PXN, were identified from COVID-19 and thrombosis datasets. Ribosomal protein S7 (rps7) is an essential component of the 40s subunit of the ribosome and is essential in the translation process and assembly of the ribosome (Fatica and Tollervey, 2002). Studies have shown that RPS7 regulates cell proliferation, the cell cycle and apoptosis through the PI3K/AKT and MAPK signaling pathways (Wang et al., 2013). However, whether RPS7 is specifically overexpressed in patients with COVID-19 with a high risk of thrombosis remains to be determined. Insulin-like growth factor 1 receptor (IGF1R) is a widely expressed membrane-bound tyrosine kinase receptor that can recognize two main ligands, IGF1 and IGF2. IGF1R expression on lymphocytes and macrophages can cause the proliferation of target cells and the production and activation of cytokines. Phosphorylation of IGF1R exacerbates inflammation, and its overexpression increases cytokine levels during influenza infection (Li et al., 2019). IGF1R has also recently been defined by some studies as a novel biomarker for predicting mortality in severe COVID-19 patients (Fraser et al., 2020). Dicer 1, ribonuclease III (DICER1) is a highly conserved RNase III ribonuclease that plays a key role in biological processes related to microRNAs (miRNAs). Recent studies have shown that DICER1 may maintain homeostasis during inflammation (Jiang et al., 2019). If the inflammatory response is out of balance, the crosstalk between inflammation and thrombosis can have serious consequences because not only does inflammation lead to thrombosis, but thrombosis in turn directly triggers inflammation. Thrombin cleaves fibrinogen and activates the cytokine IL-1α, providing a direct link between coagulation and inflammation. ERH mRNA splicing and mitosis factor (ERH) is a small protein that is highly conserved in eukaryotes. Though it was discovered nearly 30 years ago, its biological function is still largely unknown. It is considered to play a role in transcriptional regulation and the cell cycle. Although a series of studies have found that ERH has some characteristics related to RNA metabolism, the molecular functions of the ERH protein family still need to be further explored (Graille and Rougemaille, 2020). Malignant T cell-amplified sequence 1 (MCTS1) is an that was oncogene originally found in human T-cell lymphoma. MCTS1 can affect the cell cycle by shortening the G1/S conversion time, which is achieved by an imbalance between G1 cyclin activators and inhibitors. It has been confirmed that the expression of MCTS1 increases after DNA damage (Huang et al., 2021). In addition, MCTS1 also acts as a translation enhancer. Translation control has been proven to be an important step in regulating gene expression and subsequently cell growth, differentiation and necrosis (Bohlen et al., 2020). These functions enable MCTS1 to play multiple roles in the occurrence and development of malignant tumors, but its mechanisms in the process of inflammation and thrombosis have not been reported and are worth further exploration. Transportin 1 (TNPO1) is a protein involved in protein transduction into the nucleus, and its main target is RNA binding proteins. Studies have shown that TNPO1 is related to the influenza A virus core shell (Larson et al., 2019; Miyake et al., 2019). Moreover, its expression level can significantly predict ARDS mortality (Liao et al., 2021). This finding indicates that TNPO1 plays a key role in the inflammatory response and the occurrence of viral diseases, which show similar pathway activation to COVID-19 and thrombosis. During the COVID-19 pandemic, the most worrisome complications were ARDS and cardiovascular events, such as venous thromboembolism, myocardial infarction and stroke (Bangalore et al., 2020; Zhang Y. et al., 2020; Poissy et al., 2020; Stefanini et al., 2020). This theoretical evidence provides the basis for studying the mechanism of TNPO1 in COVID-19. In addition, two co-downregulated genes, FLNA and PXN, were obtained through bioinformatics analysis. Filamin A (FLNA) is a large actin-binding cytoskeletal protein. It is important for cell movement because it stabilizes the actin network and integrates it with the cell membrane. There is increasing evidence that FLNA is involved in the pathogenesis of cardiovascular and respiratory diseases. The interaction of FLNA with transcription factors and cell signaling molecules determines the function of vascular cells (Bandaru et al., 2021). Paxillin (PXN) is a focal adhesion protein consisting of an LD motif and a conserved cysteine-rich domain. PXN is thought to play a role in active muscle protein uptake and cell adhesion sites. Although PXN itself lacks enzymatic activity, it contains a variety of domains that can bind signals and structural proteins (Tsai et al., 2017). Therefore, PXN is considered to be a cytoskeletal protein that can effectively transmit signals (Stiegler et al., 2012). Inducers of these two co-downregulated genes have theoretical therapeutic value for COVID-19 patients with thrombosis. Based on the correlation between thrombosis and the severity of COVID-19 (Nicolai et al., 2020), further research may even identify a specific mechanism to reduce the rate of severe COVID-19, thereby reducing the threat of COVID-19 to the elderly and people with underlying diseases.

A limitation of our study is that these identified biomarkers were only verified at a theoretical level. Further experimental studies and clinical trials should be performed for accurate validation.

## Conclusion

Based on bioinformatics analysis and previous studies, this study identified hub genes (RPS7, IGF1R, DICER1, ERH, MCTS1, TNPO1, FLNA, and PXN) that may contribute to the evaluation and treatment of COVID-19 and thrombosis. These findings provide new ideas to treat COVID-19 thrombosis and reduce the rate of severe COVID-19, and further clinical studies are needed to explore these possibilities.

## Data Availability

Publicly available datasets were analyzed in this study. This data can be found here: https://www.ncbi.nlm.nih.gov/geo/query/acc.cgi?acc=GSE19151, https://www.ncbi.nlm.nih.gov/geo/query/acc.cgi?acc=GSE164805.

## Supplementary Material

Supplementary Figure S1 Box plots of GSE19151. The dataset included 70 adults who had used warfarin one or more times for venous thromboembolism and 63 healthy controls.

Supplementary Figure S2 Box plots of GSE164805. The dataset included 10 patients diagnosed with COVID-19 and 5 healthy controls.
