# Supplementary figures and images for: Screening the Potential Biomarkers of COVID-19-Related Thrombosis Through Bioinformatics Analysis
Source: Front Genet. 2022 May 25;13:889348. doi: 10.3389/fgene.2022.889348 (PMC9174658; doi:10.3389/fgene.2022.889348)

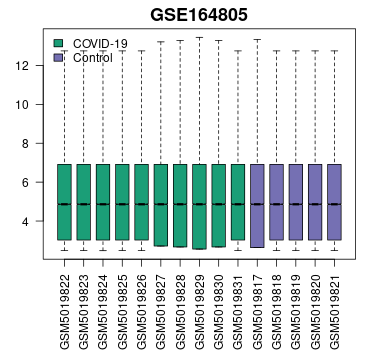

Supplement: Supplementary file 1 [file Image2.TIF]

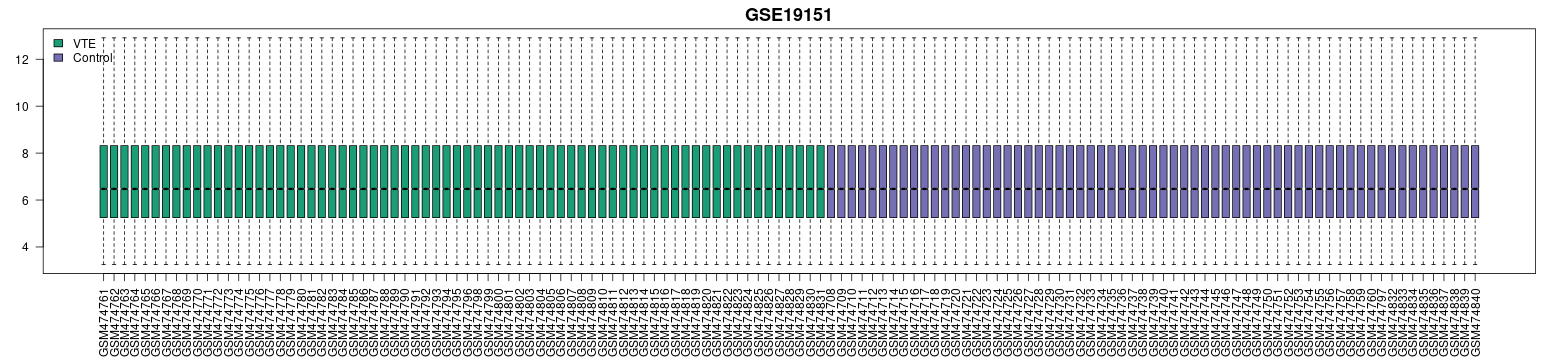

Supplement: Supplementary file 2 [file Image1.TIF]
